# A dataset of the relationship between emotional intelligence and teamwork results of university students

**DOI:** 10.1016/j.dib.2022.108149

**Published:** 2022-04-13

**Authors:** Thi-Phuong-Linh Nguyen

**Affiliations:** National Economics University, 207 Giai Phong, Hanoi, Viet Nam

**Keywords:** Emotional intelligence, Teamwork performance, University students, Vietnam

## Abstract

The dataset explores the relationship between emotional intelligence and teamwork results of university students. This study was based on a survey of 372 university students in Vietnam. Four dimensions of emotional intelligence: emotional awareness, emotion's usage, emotional understanding and emotional controlling were measured with the 18-item scale designed by Mayer and Salovey (1997) and Schutte et al. (1998). Teamwork results was assessed by using the 6-item developed by Volet and Mansfield (2006). The respondent's characteristics also explored through the survey include: gender, what year students are from, how often they work in teams. The dataset was collected to study the direct effects of emotional intelligence on teamwork performance of university students and give some recommendations to managers, lecturers and university students to promote teamwork performance of university students in general and in Vietnam in particular.

## Specifications Table

**Table:** 

Subject	Social Sciences (General)
Specific subject area	Emotional intelligence, teamwork performance, university students
Type of data	Table
How data were acquired	Survey Questionnaire (included in Supplementary Materials). Data were processed by the software SPSS 24.0 and AMOS 24.0.
Data format	Raw Analyzed
Related research article	T. H. Le, M. N. Pham, P. P. A. Nguyen, L. T. P. Nguyen, The Relationship of Emotion Intelligence, Knowledge-Sharing and Group Work Results of Vietnam Students, European Journal of Education and Pedagogy 2(3) (2021) 128–132. https://doi.org/10.24018/ejedu.2021.2.3.90.
Parameters for data collection	Participants are Vietnam university students in the economic sector (National Economics University, Banking Academy, University of Economics - National University, Foreign Trade University, Academy of Finance, University of Commerce)
Description of data collection	The data was collected directly at universities through survey forms from July to October 2020. Respondents to the survey were selected at random based on the list of universities provided. The dataset includes 372 valid responses.
Data source location	Region: Asia Country: Vietnam Latitude and longitude: 21.028511, 105.804817
Data accessibility	Data with the article

## Value of the Data

• The dataset explores the relationship between emotional intelligence and teamwork results of university students.
• The dataset applies and confirms the suitability of the inheritance of scales with university students.
• The findings of the dataset are also a good reference for both scholars and practitioners to promote the teamwork results of university students based on measures of impact on their emotional intelligence.

## 1 Data Description

The dataset explores the relationship between emotional intelligence and teamwork results of university students by applying empirical statistical methods. The initial emotional intelligence scale consisted of 33 items was designed based on the definition of Mayer and Salovey [3], Ghuman [4] and the original questionnaire by Shutte et al. [1]. A preliminary quantitative study with 20 students to check the reliability of the scales and items was done before conducting a large-scale survey. The results showed that, in the 33-item Schutte Self-Report Inventory (SSRI) developed by Schutte et al. [1], 18 items were kept and 15 items had to be removed because item-total correlations were less than 0.3 [5]. 18 items of the emotional intelligence scale were used to measure four dimensions including emotional awareness (5 items), emotion's usage (5 items), emotional understanding (5 items) and emotional controlling (3 items). The scale of teamwork results includes 6 items was proposed by Volet and Mansfield [2]. The questionnaire is provided as a supplementary file.

To collect accurate data, the author went directly to some universities in the economic sector in Vietnam to distribute and collect survey forms from July to October 2020. The survey questionnaire was divided into 2 parts: the first part explores personal information such as gender, what year students are from, and how often they work in teams; the second part to find out how felt about emotional intelligence and teamwork results. The questions are rated in a five Likert-type format ranging from 1 (strongly disagree) to 5 (strongly agree). The authors collected 385 questionnaires from university students. After screening was done, 372 questionnaires were used for the study.

Supplementary to the raw dataset describing the results of a survey of Vietnamese university students on emotional intelligence and teamwork results. Firstly, the dataset aims to provide raw data, collected from university students, to measure individuals’ self- report on four different dimensions of emotional intelligence and teamwork results. Secondly, some demographic characteristics of respondents were also included in the dataset. Finally, the dataset was used to understand the relationship between emotional intelligence and teamwork results of university students, from which a basis for making suggestions to promote teamwork results of university students through impact on their emotional intelligence. The empirical quantitative method was applied for this study with the support of software such as SPSS 24.0 and AMOS 24.0.

**Fig. 1:**
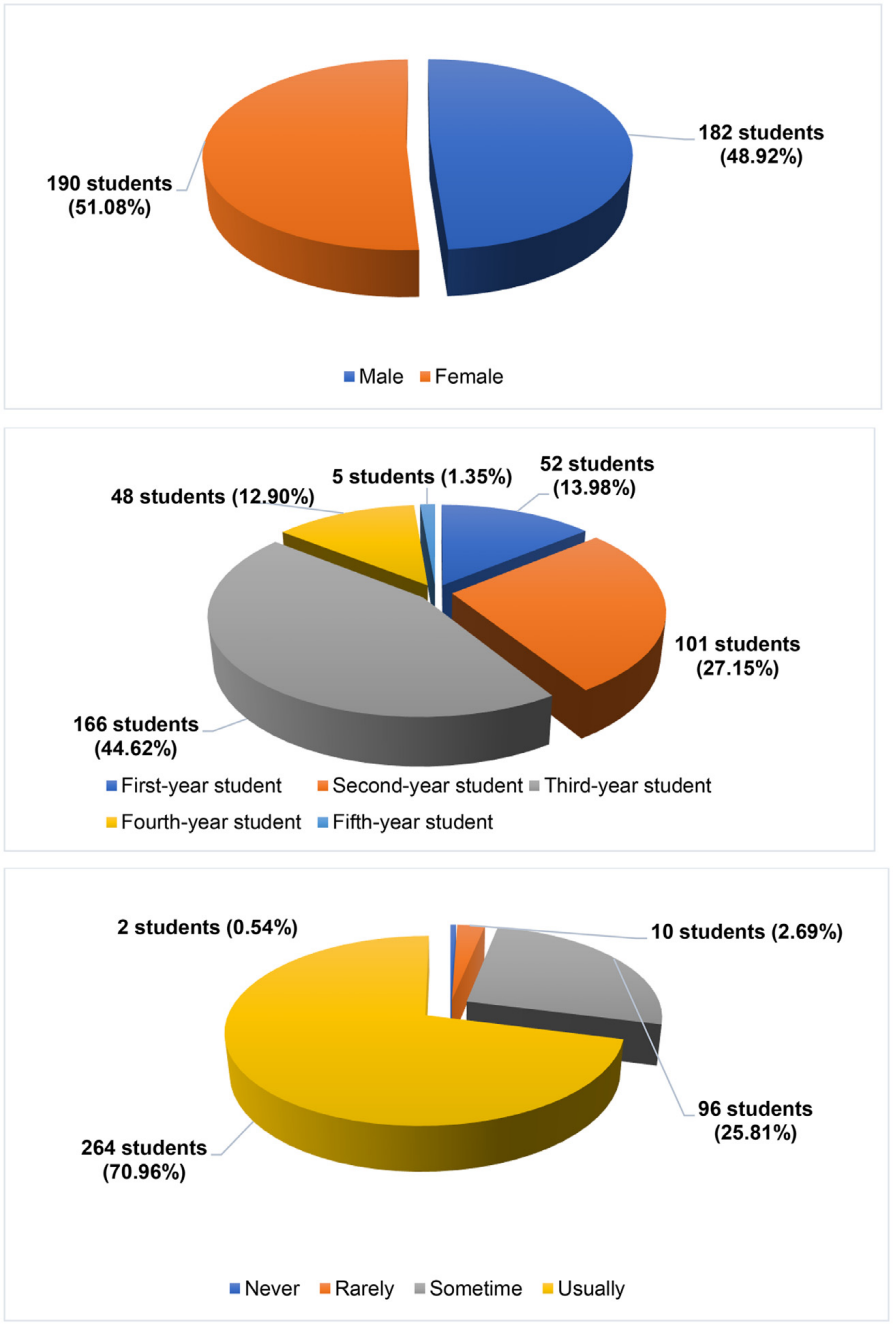
The profile of the respondents.

The first part of the questionnaire explored information about the respondents, including gender (2 categories: Male and Female), what year students are from (5 categories: First year; Second year; Third year; Fourth year and Fifth year), how often they work in teams (4 categories: Never; Rarely; Sometime; Usually). The profile of the respondents was presented in Fig. 1.

**Fig. 2:**
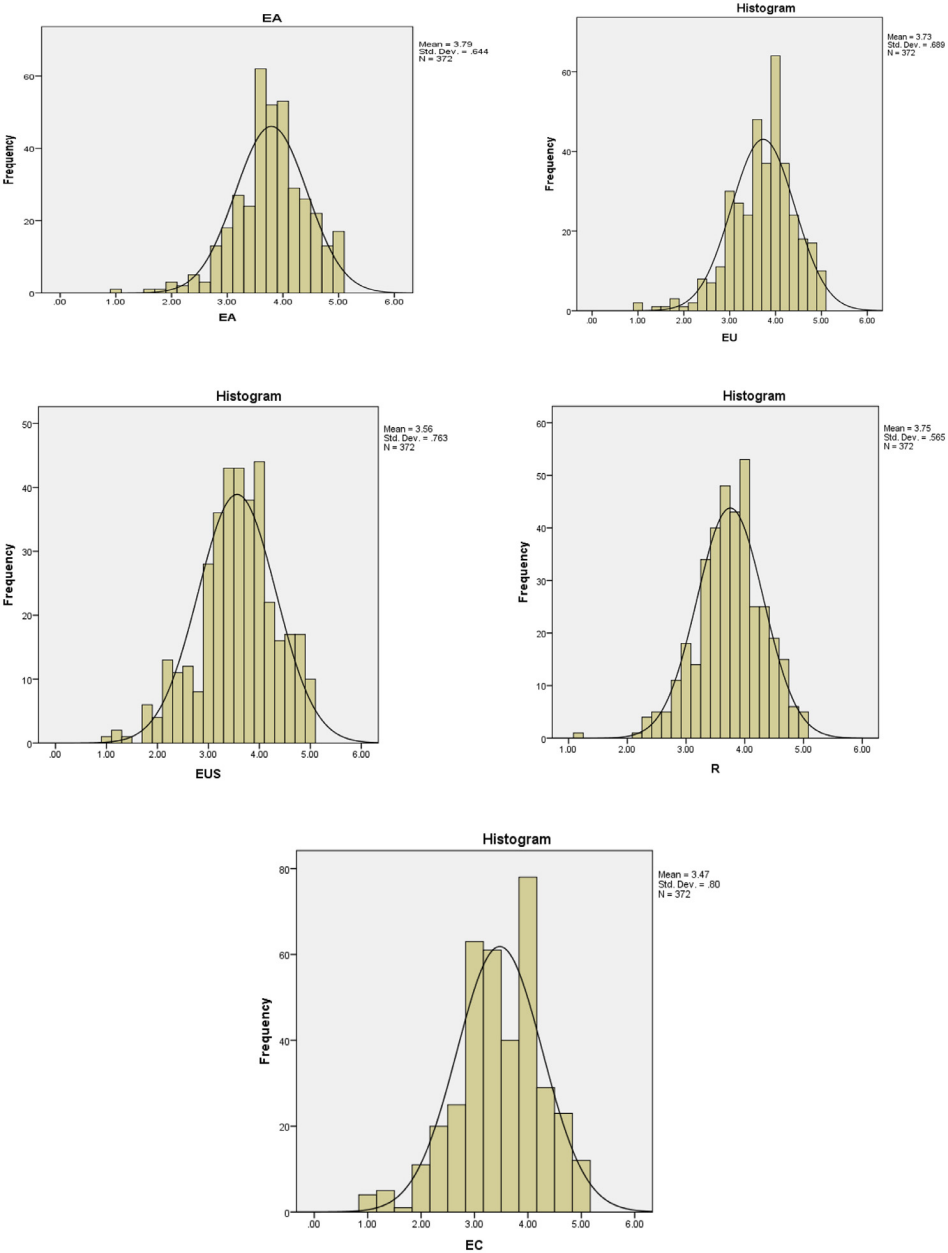
Histograms with normal curve.

The second part of the questionnaire described the degree of agreement of respondents according to the five Likert-type about emotional intelligence and teamwork results. Based on the 33-item Schutte Self-Report Inventory (SSRI) developed by Schutte et al. [1], the scale of emotional intelligence that included 18 items of four dimensions: emotional awareness (EA), emotion's usage (EU), emotional understanding (EUS) and emotional controlling (EC) with a total of 18 items. Volet and Mansfield [2] developed the scale of teamwork results that included 6 items. The results of Cronbach’ alpha and the descriptive characteristics (mean, standard deviation, skewness and kurtosis) of scales was described in Table 1. All items in the scales are within the expected range [6]. Cronbach's alpha of all variables ranges from 0.776 to 0.888 and the Corrected Item-Total Correlation of each item is higher than 0.3 [7]. All variables’ Cronbach's alpha values are acceptable for testing reliability of the scale. After analysing the reliability of scales by Cronbach's alpha, 24 items are used in the exploratory factor analysis (EFA). Testing the validity of scales by EFA (KMO = 0.888 > 0.5, Sig. (Bartlett's Test) = 0.000 〈 0.005, initial eigenvalues = 66.635 〉 50%) demonstrated that good appropriateness to perform Confirmatory Factor Analysis (CFA) [8]. EU1 was excluded from the scale because of its loading factor < 0.5 [9]. In addition, Fig. 2 illustrated the histograms with normal curve.

**Table 1:** Cronbach's alpha and descriptive characteristics of variables (*N* = 372).

Variables		Mean	SD	Skewness	Kurtosis	Cronbach's alpha
**EA**	**Emotional awareness (Schutte et al.****[1]****)**	**3.7919**	**0.64398**	**−0.508**	**1.042**	**0.865**
**EA1**	I am aware of the personal feelings when meeting someone.	4.062	0.8300	−0.912	1.194	0.855
**EA2**	I know the content/implicit that I want to convey to other members when working in a team.	3.753	0.7510	−0.481	0.554	0.821
**EA3**	I can feel and capture the emotions of other team members when talking or working with me .	3.677	0.8031	−0.104	−0.319	0.847
**EA4**	When my emotions at work change, I know clearly why I have that change.	3.726	0.8468	−0.167	−0.346	0.849
**EA5**	I feel the evaluation through the hidden meanings of the group members to me through their actions and gestures.	3.742	0.7581	−0.464	0.477	0.813
**EU**	**Emotion's usage (Schutte et al.****[1]****)**	**3.7306**	**0.68941**	**−0.713**	**1.149**	**0.776**
**EU1**	I always evaluate the importance of work and events to myself.	3.995	0.8625	−0.649	0.345	0.817
**EU2**	My ability to come up with new ideas is affected by my mood.	3.720	0.9972	−0.548	−0.070	0.723
**EU3**	My problem-solving ability/level is affected by mood.	3.626	1.0214	−0.437	−0.410	0.712
**EU4**	My responsibilities and enthusiasm for work are influenced by mood.	3.473	0.8973	−0.425	0.004	0.666
**EU5**	Emotions are one of the most meaningful things in my life because I use them all the time.	3.839	0.9606	−0.626	0.059	0.729
**EUS**	**Emotional understanding (Schutte et al.****[1]****)**	**3.5629**	**0.76318**	**−0.444**	**0.254**	**0.888**
**EUS1**	I know when to share my own problems with others.	3.651	0.9913	−0.573	0.055	0.882
**EUS2**	When communicating, I know how to arrange event content so that listeners feel comfortable.	3.497	0.9302	−0.295	−0.242	0.833
**EUS3**	When I need to express myself to someone, I always know how to make an impression on that person.	3.473	0.8605	0.020	−0.279	0.846
**EUS4**	I empathize with the stories others share with me.	3.696	0.9002	−0.366	−0.282	0.877
**EUS5**	I always believe in myself to do a good job.	3.497	0.9037	−0.355	−0.087	0.879
**EC**	**Emotional controlling (Schutte et al.****[1]****)**	**3.4722**	**0.79990**	**−0.463**	**0.308**	**0.849**
**EC1**	I know how to maintain positive emotions.	3.446	0.9985	−0.382	−0.220	0.830
**EC2**	I always create positive motivation when taking on a job.	3.543	0.9175	−0.485	0.198	0.847
**EC3**	I always control my emotions in every situation.	3.427	0.8127	−0.385	0.256	0.693
**R**	**Teamwork results (Volet and Mansfield****[2]****)**	**3.7531**	**0.56482**	**−0.409**	**0.840**	**0.876**
**R1**	My team worked together to complete the task in a timely manner.	3.927	0.7024	−0.414	0.575	0.849
**R2**	My team acted with calm and control.	3.742	0.7328	−0.423	0.580	0.851
**R3**	My team is always actively supporting each other, with a spirit of confidence, optimism and determination.	3.734	0.7606	−0.250	0.189	0.860
**R4**	My team adapts to changing situations.	3.715	0.7938	−0.518	0.306	0.859
**R5**	My team has been monitoring and reassessing the situation.	3.780	0.6807	−0.364	0.569	0.844
**R6**	My team anticipates possible scenarios.	3.621	0.6349	−0.050	−0.204	0.861

**Fig. 3:**
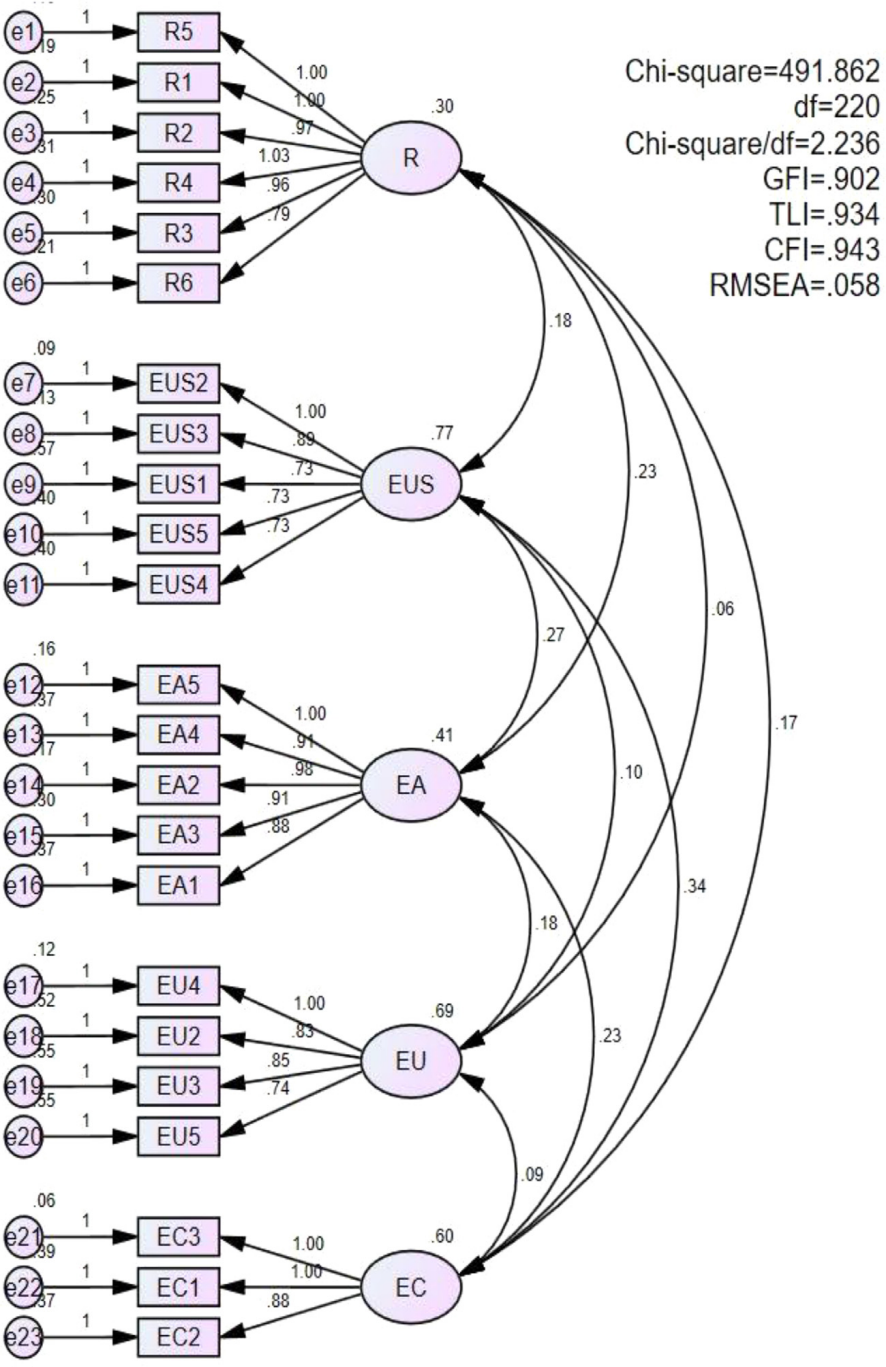
CFA results.

Confirmation factor analysis (CFA) was performed to confirm the variability of the variables in this study. The model was consistent with the data: Chi-Square (220) = 491.862; Chi-Square/df = 2.236; GFI = 0.902; CFI =0.943; TLI =0.934; RMSEA = 0.058 [6]. These CFA results confirmed satisfactory discriminatory value and showed no bias of the common method bias. The three important indicators of convergent validity are factor loadings (standardized estimates), the average variance extracted (AVE) and composite reliability (CR). The standardized estimates of each construct ranged from 0.650 to 0.957 and were statistically significant (p-values). AVE ranged from 0.546 to 0.692 and CR ranged from 0.829 to 0.893. The results of standardized estimates, AVE and CR were all in the acceptable region, thereby providing support for convergent validities of constructs [6]. The results were shown in Fig. 3 and Table 2.

**Table 2:** Standardized regression weights of items (quoted from [11]).

Constructs	Items	Estimates	Composite Reliability (CR)	Average Variance Extracted (AVE)
Emotional awareness (EA)	EA1	0.683	0.872	0.578
	EA2	0.835		
	EA3	0.726		
	EA4	0.694		
	EA5	0.848		

Emotion's usage (EU)	EU2	0.693	0.829	0.554
	EU3	0.690		
	EU4	0.924		
	EU5	0.848		

Emotional understanding (EUS)	EUS1	0.650	0.893	0.631
	EUS2	0.946		
	EUS3	0.907		
	EUS4	0.713		
	EUS5	0.712		

Emotional controlling (EC)	EC1	0.780	0.870	0.692
	EC2	0.744		
	EC3	0.957		

Teamwork results	R1	0.786	0.878	0.546
	R2	0.729		
	R3	0.695		
	R4	0.716		
	R5	0.811		
	R6	0.689		

**Fig. 4:**
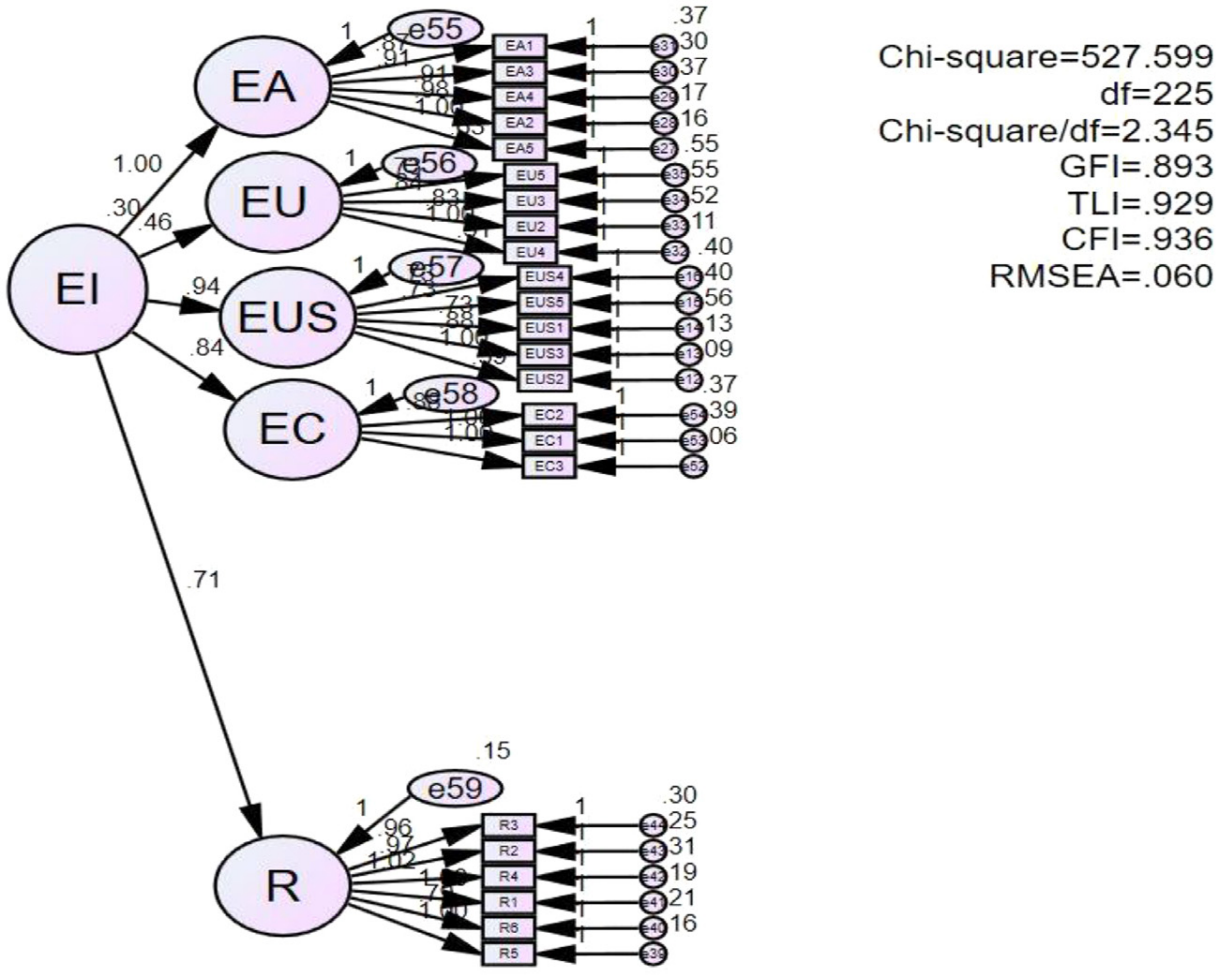
SEM results.

Structural equation modeling (SEM) was used to analyze structural relationships. SEM resluts suggested that the hypothesized model fit the data well: Chi-Square (225) = 527.599; Chi-Square/df = 2.345; GFI = 0.893; CFI =0.936; TLI =0.929; RMSEA = 0.060 [6]. In some topics, due to the limitation of sample size, it is difficult for the GFI value to reach 0.9 because this index depends a lot on the number of scales, the number of observed variables and the sample size. Therefore, if the GFI value is below 0.9 but from 0.8 or higher, it is still accepted according to studies by Baumgartner and Homburg [9] and Doll et al. [10]. The SEM results described in detail in Fig. 4.

## 2 Experimental Design, Materials and Methods

To collect accurate data, the author went directly to universities in the economic sector (National Economics University, Banking Academy, University of Economics - National University, Foreign Trade University, Academy of Finance, University of Commerce) in Hanoi to distribute and collect survey questionnaires from July to October 2020. The author asked each university to send a list of 150 students. Then, the author randomly selected 60–70 students from each university based on the list and made an appointment to meet in a lecture hall of the university itself. Each student took about 15 min to complete the survey. Total number of questionnaires distributed was 385 questionnaires, the number of questionnaires collected was 380, the number of questionnaires collected after cleaning was 372, estimated at 96.6%.

The survey questionnaire is divided into 2 parts: the first part to find out how respondents felt about emotional intelligence and teamwork performance; the second part explores personal information such as gender, what year students are from, and how often they work in teams.

The survey was designed with 27 items, of which 3 items were about the characteristics of the respondents, the remaining 24 items were designed on a 5-point Likert scale (1: Strongly disagree; 2: Disagree; 3: Neutral; 4: Agree; 5: Strongly agree), focusing on 2 factors: (1) emotional intelligence; (2) teamwork performance. All items in the survey were inherited from previous studies [1,2]. The questionnaire was only valid when fully filled in both parts of the questionnaire. After removing invalid questionnaires, the final dataset contains 372 questionnaires. All respondents' responses were coded and entered into Excel software before being imported into SPSS 24.0 and AMOS 24.0.

Based on the data set, further researches can study the direct effects of emotional intelligence on teamwork performance of university students and give some recommendations to managers, lecturers and university students to promote teamwork performance of university students in Vietnam.

## Ethics Statement

The author kept to all ethical concerns during the data gathering process. The authors got the consent of the response when conducting surveys. The author has received the consent of the collaborators for the use of collected data to complete the paper.

## CRediT authorship contribution statement

**Thi-Phuong-Linh Nguyen:** Conceptualization, Methodology, Software, Formal analysis, Data curation, Writing – original draft, Writing – review & editing, Funding acquisition.

## Declaration of Competing Interest

The author declare that they have no known competing financial interests or personal relationships which have, or could be perceived to have, influenced the work reported in this article.

## Data Availability

Emotional intelligence and teamwork results of university students (Original data) (Mendeley Data).
